# Photothermal effects of gold nanorods in aqueous solution and gel media: Influence of particle size and excitation wavelength

**DOI:** 10.1049/nbt2.12110

**Published:** 2022-12-21

**Authors:** Zendesha S. Mbalaha, David J. S. Birch, Yu Chen

**Affiliations:** ^1^ Department of Physics Scottish Universities Physics Alliance University of Strathclyde Glasgow UK; ^2^ Department of Science Education Joseph Sarwuan Tarka University Makurdi Benue State Nigeria

**Keywords:** gold nanorods, photothermal effect, photothermal therapy, surface plasmon

## Abstract

Gold nanorods (GNRs) have emerged as the most efficient photothermal agent in cancer therapy and photocatalysis. Understanding the influence of the surrounding medium, particle size, and excitation wavelength is critical to optimising the photothermal conversion rate. Here, three pairs of large and small gold nanorods of different aspect ratios and their heat generation under laser radiation at on and off surface plasmon resonance wavelengths in aqueous solution and gel‐like media are investigated. In the aqueous solution, the temperature rise of the large gold nanorods is more than with small gold nanorods at resonance excitation. In contrast to the large gold nanorods (LGNRs), the small gold nanorods (SGNRs) were less sensitive to excitation wavelength. At off‐resonance excitation, the temperature rise of the SGNRs is larger than that of the LGNRs. In the agarose gel, the photothermal effect of the SGNRs is greater than LGNRs excited at the wavelength near their solution phase longitudinal surface plasmon resonance wavelength. The temperature increase of LGNRs in gel is significantly less than in aqueous solution. These findings suggest that SGNRs could be more beneficial than the LGNRs for photothermal applications in biological systems and provides further insight when selecting GNRs.

## Graphical abstract

Photothermal effect of large and small gold nanorods of different aspect ratios have been investigated at on and off surface plasmon resonance wavelengths in solution and gel media. In contrast to large gold nanorods, small gold nanorods were less sensitive to the excitation wavelength. This leads to more enhanced heat generation of small gold nanorods in gel media as surface plasmon resonance wavelength shifts when gold nanorods change media from water to gel.

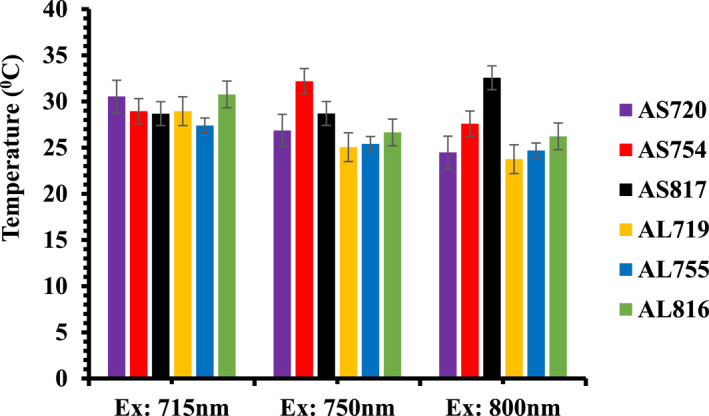

## 1 INTRODUCTION

Gold nanorods (GNRs) have attracted a great deal of attention because of their excellent size and shape‐dependent optical properties occasioned by localised surface plasmon resonance and these properties have been exploited for biomedical applications such as photothermal therapy of cancer, dark field cell imaging, and remote release of drugs [1, 2, 3, 4, 5]. The longitudinal surface plasmon resonance (LSPR) of GNRs varies with particle size, aspect ratio, and dielectric constant of the surrounding medium [1, 6]. The LSPR enhances light absorption and scattering efficiency a million times more intense than organic dyes (e.g. indocyanine green and porphyins) [1, 5, 7]. By a laser illumination, the excited free electrons in the gold nanorods oscillate coherently in resonance with the incident light leading to a strong absorption and scattering of light from the visible to the near infrared wavelengths of the electromagnetic spectrum where the biological tissue transmission efficiency is very high [5, 8, 9]. Moreover, GNRs are biocompatible and photostable, and have a longer period (∼17 h) of circulation in the blood than other gold nanoparticles (nanospheres, nanoshells, and nanocages) due to their anisotropic shape [5, 10]. These excellent attributes of the GNRs informed the choice of GNRs for this study to gain further insight into the influence of medium, size of GNRs and excitation wavelength on the photothermal effect of GNR in order to optimise their performance in biomedical applications.

The photothermal energy of GNRs can be achieved through photo‐physical processes following the absorption of incident light. That is, upon optical absorption, the excited free electrons in GNRs interact with neighbouring electrons via electron‐electron collisions generating hot electrons with a temperature of about 1000K in femtoseconds. The hot electrons dissipate their energy to the particle lattice via electron‐phonon relaxation in 0.5–1 picoseconds resulting in a hot lattice with a temperature rise on the order of a few tens of degrees. The particle lattice cools off by dissipating its heat energy to the surrounding medium via phonon‐phonon relaxation in about 100 picoseconds [1, 5, 11, 12]. Previous studies have investigated the influence of morphology of gold nanoparticles on heat generation [13, 14], and found that gold nanorods generate heat ∼6 times faster than the gold nanospheres (GNSs) [5, 14]. In addition, Yang et al. [15]. found that gold nanostars (GNSTs) have better photothermal performance in contrast to GNRs and GNSs; however, the hydrodynamic size of GNSTs is larger than GNRs and GNSs, which may affect cell uptake and migration through biological barriers. Jia et al. [2] have reported that the optical extinction of small gold nanorods (SGNRs) is dominated by optical absorption, thus, the SGNRs have a superior photothermal effect compared to the large gold nanorods (LGNRs). Furthermore, a comparison of the photothermal effect of different size of GNRs under a single excitation wavelength revealed that though the small size GNRs have larger absorption/extinction ratio and larger photothermal conversion efficiency, the large size GNRs have a stronger heating effect of the entire aqueous solution due to a stronger field coupling [11].

The photothermal effect of GNRs in cancer therapy has been demonstrated in various studies. For example, Manivasagan et al. demonstrated that cancer cells in cell culture and animal model treated with gold nanorods could be killed due to the photothermal effect of functionalised gold nanorods [3]. Bucharskaya et al. showed that increasing the concentration of gold nanorods in tumour cells rises the local temperature of tumour environment thereby suppressing tumour growth and eventual death [16]. Human glioblastoma (U87MG) cells treated with functionalised gold nanorods‐graphene oxide nanocomposite were destroyed upon irradiation by a continuous wave laser [4].

The localised surface plasmon resonance of GNRs is very sensitive to the surrounding medium [6, 17], thus, the photothermal effect of GNRs could vary in different media [18]. Anderson et al. noted that surface plasmon resonance of GNSs embedded in gold‐silica aerogels blueshifts relative to the same GNSs in gold solution due to their interaction with the gel network [19]. Lazar et al. found that gold nanoparticles could undergo structural changes and loss of surface plasmon absorption due to the formation of larger aggregates in different media [18]. Moretti et al. investigated the drug release efficiency of PEGylated and non‐PEGylated GNSs [20]. The PEGylated GNSs were stable and dispersed in agarose‐loaded hydrogel while the non‐PEGylated GNSs formed aggregates in the hydrogel. The photothermal effect of non‐PEGylated GNSs was higher than that of PEGylated GNSs because of the stronger coupling effect of the aggregators. When mimicking drugs were mixed with the hydrogel, it was found that the drugs released efficiency of the non‐PEGylated drugs was higher relative to that of the PEGylated GNSs. Yang et al. investigated the chemo‐photothermal effect of GNRs immobilised with an extracellular matrix mimicking gel known as nanogel derived from a polypetide [13]. The nanogel was adsorb on the surface of GNRs and doxorubicin (DOX, a cancer drug) was introduced onto the nanogel. The drug release rate of GNR‐nanogel‐DOX complex exposed to laser illumination was faster and higher in comparison to the same complex without exposition to laser illumination. Furthermore, in vitro studies revealed that Hela cell treated with GNR‐nanogel‐DOX complex and laser illumination died more frequently than the Hela cell treated with laser alone, and the Hela cells with GNR‐nanogel‐DOX complex alone [21], thus, revealing the efficacy of synergistic therapy in cancer management. Lee et al. [22] investigated the influence of particle shape on diffusion in a gel media. Polystyrene nanospheres were stretched mechanically to a nanorod shape. It was observed that the nanorod‐shape particle incorporated in a composite hydrogel diffuse faster relative to the nanospheres. Additional studies on the photothermal effect of GNRs are documented in Table 1.

**TABLE 1:** Studies on the photothermal effect of GNRs

Authors	Particle shape	Medium	Excitation (nm)	Year	Ref.
Pratap et al.	SGNRs	Water		2022	[23]
Gong et al.	GNRs	Cell	808	2021	[24]
Moretti et al.	GNSs	Water	400	2021	[20]
Meyer et al.	LGNRs and SGNRs	Water	754, 761, 848, 886	2021	[25]
Awan et al.	SGNRs	Water and cell	808	2021	[26]
Bermúdez‐jiménez et al.	GNRs	Bacteria	810	2019	[27]
Yang et al.	LGNRs	Polypeptide based nanogel	810	2017	[28]
Qin et al.	GNRs and GNSs	Water	Unspecified	2016	[13]
Mackey et al.	LGNRs and SGNRs	Water and cell	808	2014	[11]
Wang	GNRs, GNSs, and GNSTs	Water	785	2014	[29]
Canpean et al.	GNRs	Water	Unspecified	2013	[30]

Abbreviations: GNR, gold nanorod; GNSs, gold nanospheres; GNST, gold nanostar; LGNR, large gold nanorod; SGNR, small gold nanorod.

The heat generated by GNRs can be influenced not only by the particle size, but also by the surrounding medium and excitation wavelength [31]. However, so far a single wavelength near surface plasmon resonance wavelength of GNRs was used in the study of photothermal effect of GNRs [11, 32]. It is not clear how the different media and excitation wavelengths affect the heat generation of GNRs of different sizes. Hence the motivation for the present work.

Herein, we investigated the photothermal process of LGNRs and SGNRs of three selected aspect ratios at both on and off resonance excitation wavelengths in solution and gel media. Such a study is an important step for understanding the photothermal effect of GNRs in cells. It was found that while the large GNRs have larger temperature increase on resonance illumination than the small GNRs in solution, the small GNRs are less sensitive to the excitation wavelength. Furthermore, in the agarose gel, the small GNRs show more enhanced photothermal effect than the large GNRs due to the shift of surface plasmon resonance wavelength of GNRs resulting from the change of media. These findings suggest the importance of considering the surrounding medium, size and excitation wavelength to optimise the photothermal efficiency of GNRs.

## 2 EXPERIMENTAL SECTION

### 2.1 Materials

All chemicals were used as received without further purification. Chloroauric acid (HAuCl_4_, 49%), hexadecyltrimethylammonium bromide (CTAB, 99%), ascorbic acid (AA), sodium borohydride (NaBH_4_, 99.8%), silver nitrate (AgNO_3_) were all purchased from Sigma Aldrich while hydrochloric acid (HCl) was purchased from Fluka.

### 2.2 Synthesis of gold nanorods

Both the LGNRs and the SGNRs were synthesised using modified silver‐assisted seed mediated growth methods. The LGNRs were synthesised according to a reported protocol [33] with the volume of reagents scaled down as presented in Table S1. SGNRs were synthesised by our modified silver‐assisted seed growth method [34] and the list of chemicals for the synthesis of SGNRs are presented in Table S2 together with the description of the synthesis method.

### 2.3 Sample preparation and experimental setting for photothermal study in the water

**FIGURE 1:**
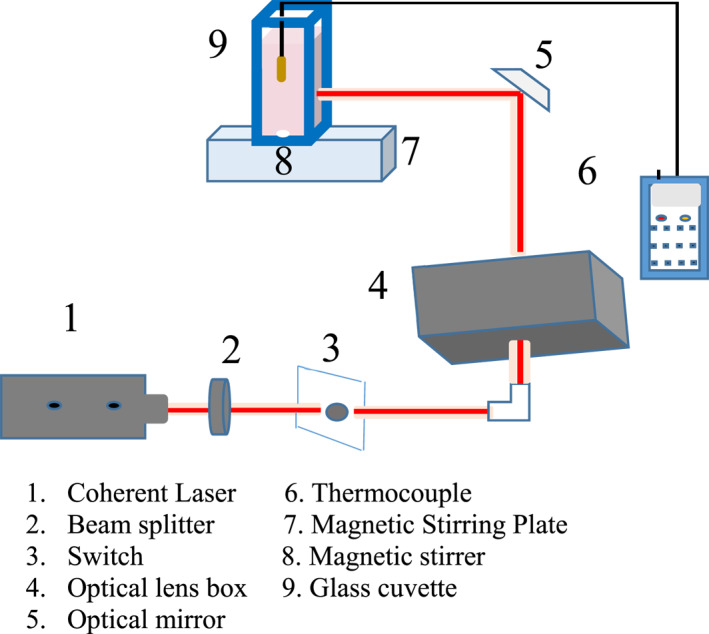
The experimental set up for laser excitation of gold nanorods.

The synthesised LGNRs and SGNRs were centrifuged at room temperature at 13,000 rpm for 15 min and the optical density (0.99) of both the colloids of the LGNRs and the SGNRs in 3.3 ml of water in a cuvette were determined with a UV‐vis spectrophotometer. Both colloids of GNRs were at room temperature (19.80°C) before excitation in a glass cuvette at 715, 750, and 800 nm excitation wavelengths using a femtosecond Ti: Sapphire laser (Chameleon, Coherent, Santa Clara, California) for 16 min. The laser pulse has a repetition rate of 80 MHz and duration of less than 200 fs. A magnetic stirrer and a thermocouple (Omega, HH804) were both inserted into the glass cuvette to ensure uniform distribution of heat and record the temperature rise respectively. The depth of the thermocouple's probe was maintained at the same position (1.4 cm) for each measurement from the top of the cuvette. The excitation beam was propagated perpendicular to the glass cuvette containing colloids of SGNRs and LGNRs. A coherent laser power metre (FieldMax_ll_) was used to measure the incident power density. The experimental set up for photoexcitation is shown in Figure 1.

### 2.4 Sample preparation for photothermal effect study in the agarose gel

The concentration of agarose gel commonly used for biological applications ranges from 0.5% to 2% [35]. This is because an agarose gel possesses physicochemical characteristics similar to the cellular cytoplasm and the biological fluids in this range [36]. Thus, 0.7% concentration of agarose gel was chosen. Firstly, agarose (0.7%, 0.14 g) powder was dissolved in 20 ml of TBE buffer at 70°C and allowed to cool at room temperature. Then, 3.3 ml of agarose gel was placed into a plastic cuvette. The pellets of the SGNRs and the LGNRs were cast into the 3.3 ml of agarose gel while the UV‐vis extinction spectra were measured until the extinction was raised to an optical density of 0.99. Thereafter, the 3.3 ml of agarose‐gold nanorods mixture were transferred to a glass cuvette and excited under similar experimental condition as above.

### 2.5 Optical and morphological characterisation of gold nanorods

The extinction spectra of the GNRs samples were measured with a UV‐vis spectrophotometer (Lambda 2, Perkin Elmer). The sizes of the LGNRs and the SGNRs were determined by SEM imaging (SEM, FEI Quanta FEG 250).

## 3 RESULTS AND DISCUSSION

### 3.1 The extinction spectra of GNRs

**FIGURE 2:**
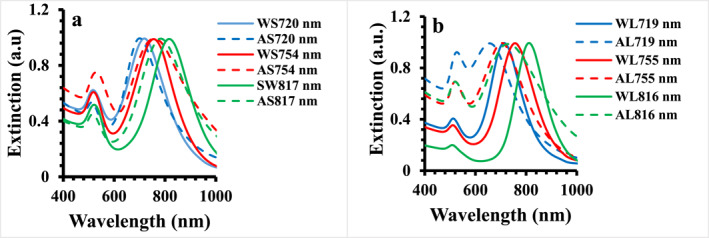
The UV‐vis extinction spectra of gold nanorods in the water and agarose gel media (0.7%); (a) the UV‐vis extinction spectra of SGNRs, (b) the UV‐vis extinction spectra of LGNRs. W and A represent sample in the water and agarose gel media respectively.

Figure 2 shows the extinction spectra of SGNRs and LGNRs in aqueous solution featuring the longitudinal surface plasmon bands centred at 720, 754 and 817 nm for three SGNRs, and 719, 755, and 816 nm for three LGNRs respectively. The samples were designated as S720, S754, S817, L719, L755, and L816. The average dimensions of these GNRs extracted from the SEM images of over 100 particles are listed in Table 2 (Typical SEM images of S817 and LGNRs L816 are displayed in the Figure S1a,b). In the agarose gel, the longitudinal surface plasmon band of the S720, S754, and S817 blue shifted to 701, 749, and 785 nm, while L719, L755, and L816 blue shifted to 657, 704, and 730 nm respectively. The longitudinal surface plasmon resonance of gold nanorods depends on the shape of the particle and refractive index of medium [1, 6]. The blue shift observed in agarose gel is likely due to the change of refractive index of medium, although the influence of reduced aspect ratio cannot be excluded.

**TABLE 2:** The longitudinal absorption, length, width and aspect ratio of SGNRs and LGNRs

Samples	LSPR (nm)	Length (nm)	Width (nm)	Aspect ratio
S720	720	19.7 ± 4.3	5.5 ± 1.3	3.6
S754	754	20.9 ± 4.5	5.6 ± 1.3	3.7
S817	817	24.0 ± 6.5	6.0 ± 1.0	4.0
L719	719	41.1 ± 6.8	11.2 ± 2.2	3.7
L755	755	44.5 ± 7.1	11.4 ± 2.0	3.9
L816	816	46.2 ± 8.4	11.5 ± 1.5	4.0

Abbreviations: LGNR, large gold nanorod; LSPR, longitudinal surface plasmon resonance; SGNR, small gold nanorod.

### 3.2 The temperature profiles of SGNRs and LGNRs illuminated in the water suspension

**FIGURE 3:**
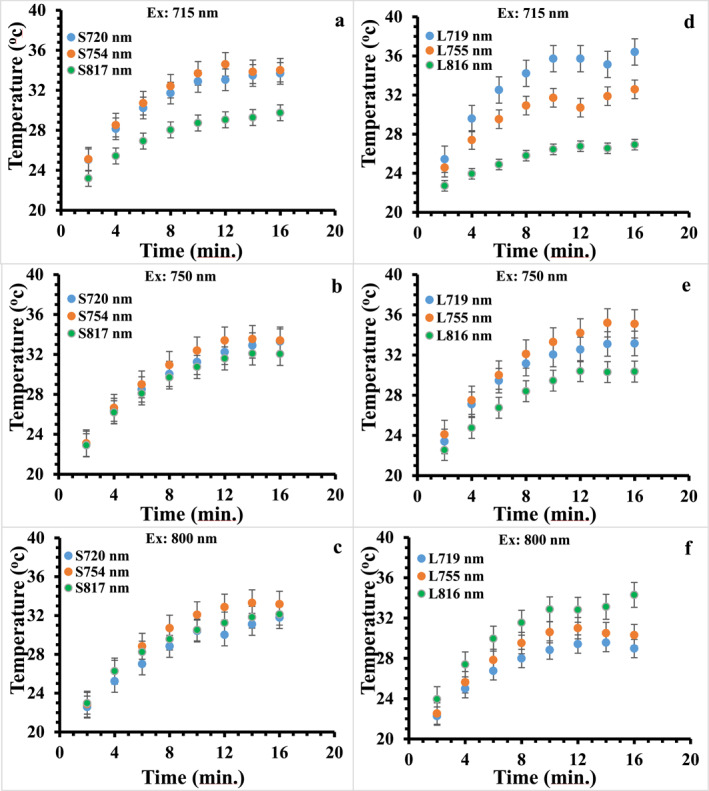
The time‐dependent temperature changes of the SGNRs and the LGNRs after laser illumination in water; (a–c) SGNRs under excitation at 715, 750, and 800 nm; (d–f) LGNRs under excitation at 715, 750, and 800 nm. The temperature profiles are normalised against their laser intensities. LGNR, large gold nanorod; SGNR, small gold nanorod.

To investigate the influence of medium and laser wavelength on the temperature response of the SGNRs and the LGNRs in the water solution and agarose gel, three laser wavelengths were chosen, namely 715, 750, and 800 nm, as they overlapped with the longitudinal surface plasmon resonance of S720/L719, S754/L755, and S817/L816 respectively. The temperature responses of the SGNRs and the LGNRs were normalised against 2.74 ± 0.16 W/cm^2^ laser intensity because the laser intensities at 715 and 800 nm laser wavelengths were 2.57 ± 0.15 and 2.78 ± 0.05 W/cm^2^ respectively, while the laser intensity at 750 nm laser wavelength was 2.74 ± 0.16 W/cm^2^. The extinction spectra of SGNRs and LGNRs before and after laser irradiation are displayed in the Figure S2. It can be seen that the temperature of all the samples increased with irradiation time as shown in Figure 3 and there is an initial fast rise up to 10 min, followed by a slow increase before reaching saturation. The temperature profiles of SGNRs under 750 and 800 nm irradiation were overlapped, same as the temperature profiles of S720 and S754 under 715 nm irradiation. The similar temperature profiles of the SGNRs on resonance excitations and off‐resonance excitations, is likely due to the broad surface plasmon bands and slight mismatch of the irradiation wavelength with the surface plasmon resonance wavelength. For example, the extinction of S754 is close to that of S720 and both are clearly larger than the extinction of S817 at 715 nm, while a larger extinction results in higher temperature rise [5]. However, the surface plasmon enhancement effect was apparent for the LGNRs where temperature increase was larger at surface plasmon resonance wavelength than that at off‐resonance wavelength.

**FIGURE 4:**
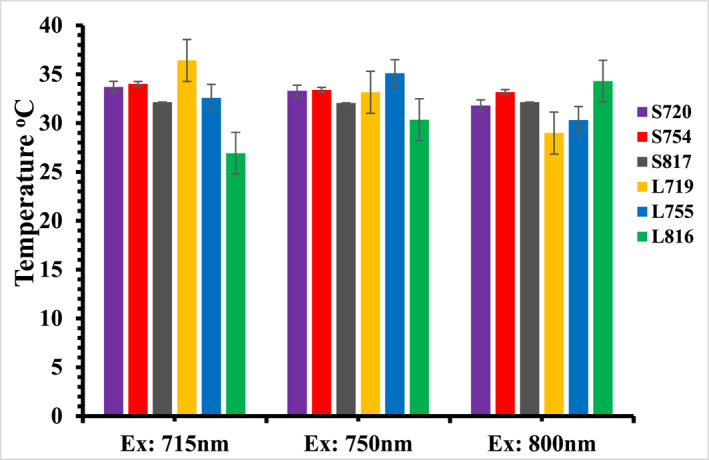
A comparison of the saturation temperature taken at 16 min of the SGNRs and the LGNRs colloid at resonance and off resonance excitations in solution. LGNR, large gold nanorod; SGNR, small gold nanorod.

Figure 4 compares the saturation temperature (taken at 16 min) of SGNRs and LGNRs excited at resonance and off resonance wavelengths. It can be seen that the temperatures of L719, L755, and L816 are higher than their SGNRs counterparts when they were excited near their corresponding resonance excitations. It was reported that the field coupling between neighbouring GNRs generates a strong electric field that enhances the heating of the GNRs solution [7, 11, 37, 38]. Despite a relatively strong absorption, SGNRs have a shorter field length. On the other hand, the LGNRs have an electric field extending further away from the surface of the GNRs. Thus, the field coupling of the LGNRs is stronger than that of the SGNRs at plasmon resonance excitation [11, 39]. This could explain the slightly higher temperature rise in the solution of the LGNRs compared to the solution of the SGNRs observed in Figure 4.

In contrast to the on‐resonance excitation, we observed that the temperature of the SGNRs increased more than that of the LGNRs at the off‐resonance excitation. This is possibly because the absorption/scattering ratio of small GNRs is higher than that of large GNRS and the contribution of field coupling to the heating process is negligible due to a weak local electric field for both small and large GNRs at off‐resonance excitation. This observation indicates the importance of excitation laser wavelength when comparing heat generation of SGNR and LGNR.

### 3.3 The temperature profile of SGNRs and LGNRs illuminated in the agarose gel

**FIGURE 5:**
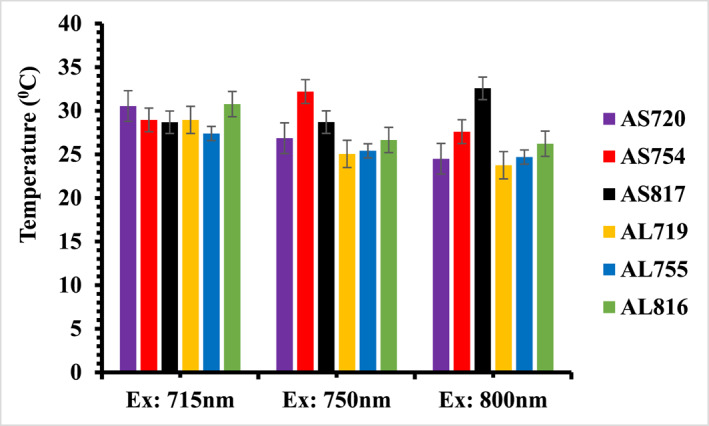
A comparison of the saturation temperature taken at 16 min of the SGNRs and the LGNRs colloid at resonance and off resonance excitations in agarose gel. LGNR, large gold nanorod; SGNR, small gold nanorod.

Figure 5 compares the saturation temperature (taken at 16 min) of the gold nanorods in the TBE buffer agarose gel illuminated at 715, 750 and 800 nm laser wavelengths. The temperature profiles of the SGNRs and the LGNRs in agarose gel were normalised against 2.74 ± 0.16 W/cm^2^ laser intensity as explained above. The saturation temperatures of the SGNRs in agarose gel, AS720, AS754, and AS817, are higher at excitation wavelengths of 715, 750, and 800 nm respectively as their LSPR partially overlaps with the excitation wavelength leading to stronger absorption of light than that at off resonance excitations. However, no obvious surface plasmon (SP) enhancement of temperature was observed for the LGNRs in agarose gel (AL719 and AL755.) except AL816 whose temperature is slightly higher than that of the AL755 and the AL719 at 800 nm illumination. This is not surprising as larger blue shifts were observed for LGNRs in agarose gel than those of SGRNs. Light absorption decreases when illumination wavelength is away from the surface plasmon resonance wavelength, resulting in reduced heat generation [40].

**FIGURE 6:**
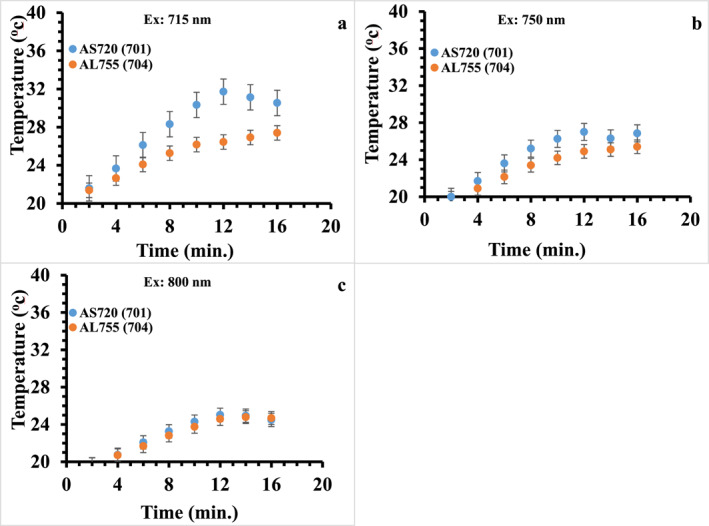
A comparison of the temperature profile of the ASGNRs and the ALGNRs with similar LSPR at; (a) 715 nm, (b) 750 nm, and (c) 800 nm illuminations. ALGNR, large gold nanorods in agarose gel; ASGNR, small gold nanords in agarose gel; LSPR, longitudinal surface plasmon resonance.

Figure 6 compares the temperature changes of the AS720 and the AL755 with similar LSPR (701 and 704 nm respectively) at 715, 750, and 800 nm laser illumination. It can be seen that the temperature of the AS720 sample is more enhanced than that of the AL755 sample at 715 and 750 nm laser illuminations. In comparison to the AL755, AS720 shows a faster temperature rise from 2 min until reaching saturation temperature after 12 min. This could be due to a higher absorption to extinction ratio of the SGNRs compared to the LGNRs at 715 and 800 nm, thus a larger heat generation. It is found that the saturation temperature of the AS720 decreases from 31°C (Ex: 715 nm) to 27°C (Ex: 750 nm) and finally 24°C (Ex: 800 nm) as the wavelength of the laser moves away from its LSPR. Moreover, the temperature difference between the two samples also decreases as the illumination wavelength increases and both temperature profiles overlap at 800 nm illumination. This is due to a similar absorption of both particles as AS720 has a smaller extinction coefficient than that of AL755 at 800 nm but a higher absorption to extinction ratio resulting in a similar heat generation.

### 3.4 The influence of the surrounding medium on the photothermal effect of GNRs

**FIGURE 7:**
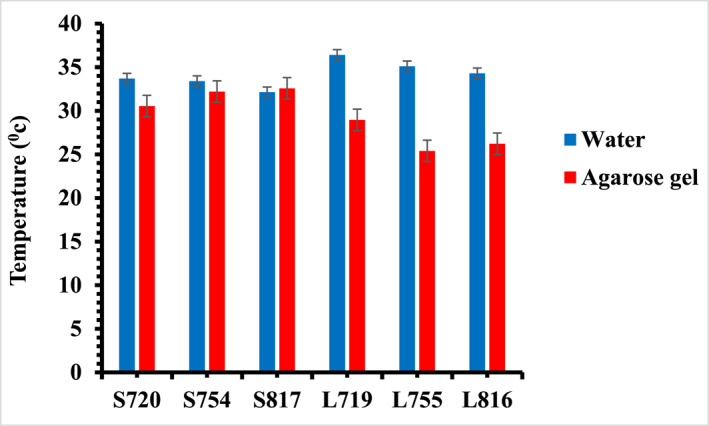
A comparison of the temperature of SGNRs and LGNRs in the water and agarose gel. LGNR, large gold nanorod; SGNR, small gold nanorod.

The influence of the surrounding medium on the photothermal effect of GNRs was investigated by comparing GNRs suspended in solution and agarose gel media. Figure 7 shows the temperature attainment of GNRs at 16 min of photoexcitation in aqueous solution and agarose gel media. The temperature attained by SGNRs in the agarose gel is higher than that of LGNRs at on‐resonance excitation, different from that in the solution. In addition, the temperature of the SGNRs (AS720 and AS754) in the agarose gel slightly decreases than that in the solution while that of AS817 is similar in both media. However, the temperature of the LGNRs in the agarose gel decreases significantly than that in the solution. The significant decrease of the temperature of LGNRs in the agarose gel is due to the large blue shift of surface plasmon resonance wavelength arising from the change of medium, resulting in reduced absorption and heat generation [41, 42, 43].

**FIGURE 8:**
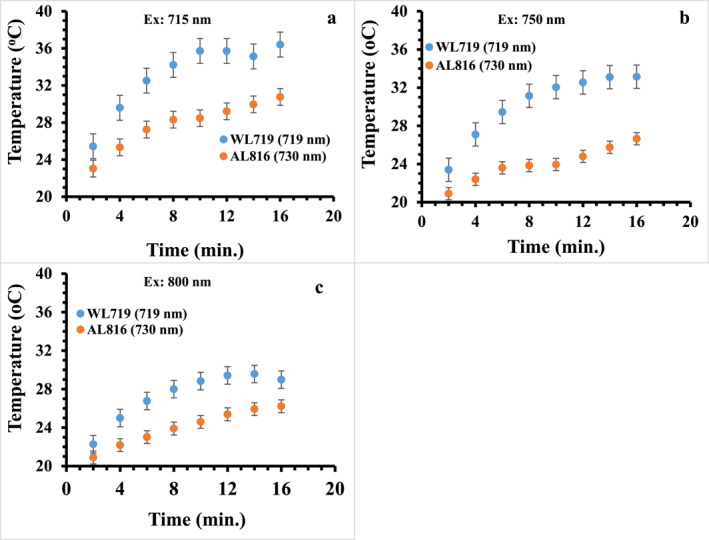
A comparison of the temperature of the WL719 colloids in the water with the AL816 in the TBE buffer‐agarose gel at varying excitation wavelengths; (a) Ex: 715 nm, WL719/AL816, (b) Ex: 750 nm WL719/AL816 and (c) Ex: 800 nm, WL719/AL816.

Indeed, AL816 has a SP centred at 730 nm that is comparable to the SP of WL719 at 719 nm. Figure 8 compares the temperature profile of the WL719 in the water with that of the AL816 in the TBE buffer‐agarose gel at 715, 750 and 800 nm excitation wavelengths. It can be seen that sample WL719 shows a faster temperature rise from 2 min until saturation temperature is reached after 12 min. Apparently, the temperature of WL719 in solution is higher than that of the AL816 in the TBE buffer‐agarose gel at 715, 750, and 800 nm laser illuminations. As observed before, the LGNRs in the solution have large electric field coupling under light excitation that could be reduced in the gel because the LGRNs are less mobile in the gel matrix. In addition, the heat transfer efficiency of the gel is reduced compared to that in the solution. It has been reported previously that gels reduce the efficiency of heat transfer because of the limited contact of granules in the gels [41, 42]. Moreover, the thermal conductivity of the agarose gel (0.55 W/m °C at 20–80°C) is slightly less than that of the water (0.6 W/m °C at 20°C) [43, 44], implying a reduced heat transfer of the agarose gel in comparison to the water solution.

## 4 CONCLUSION

The understanding of photothermal effects of SGNRs and LGNRs in a mimicked cellular microenvironment is essential for their photothermal applications. This work investigated the photothermal effects of three pairs of SGNRs and LGNRs with near identical longitudinal surface plasmon resonance wavelength under on‐resonance and off‐resonance excitation in the aqueous solution and agarose gel. In aqueous solution, the temperature rise of the LGNRs is more enhanced than that of the SGNRs at the plasmon resonance excitation, while at off resonance excitation, the temperature rise of the SGNRs is more enhanced than that of the LGNRs. Moverover, wavelength dependence is less significant in the case of SGNRs. In agarose gel, the temperature rise of the SGNRs is more enhanced than that of LGNRs when they are excited at the wavelength near their LSPR wavelength in solution. The temperature rise of LGNRs in aqueous solution is larger than that in agarose gel. This is due to the surface plasmon wavelength shift resulting from the change of medium. These findings suggest the importance of considering the influence of the surrounding medium and laser wavelength when studying size effect of GNRs in heat generation. The SGNRs could be more beneficial than the LGNRs for photothermal applications in biological systems, thus, providing further insight in selecting GNRs for efficient photothermal applications.

## AUTHOR CONTRIBUTIONS

**Zendesha S. Mbalaha**: Investigation; Writing – original draft. **David J. S. Birch**: Supervision; Writing – review & editing. **Yu Chen**: Conceptualisation; Funding acquisition; Methodology; Project administration; Resources; Supervision; Writing – review & editing.

## CONFLICT OF INTEREST

There is no conflict of interest.

## Supporting information

Supporting Information S1

## Data Availability

The data that support the findings of this study are available from the corresponding author upon reasonable request.

## References

[1] Huang, X. , El‐Sayed, M.A. : Gold nanoparticles: optical properties and implementations in cancer diagnosis and photothermal therapy. J. Adv. Res. 1, 13–28 (2010)

[2] Jia, H. , et al.: Synthesis of absorption‐dominant small gold nanorods and their plasmonic properties. Langmuir 31(26), 7418–7426 (2015). 10.1021/acs.langmuir.5b01444 26079391

[3] Manivasagan, P. , et al.: Chitosan/fucoidan multilayer coating of gold nanorods as highly efficient near infrared photothermal agents for cancer therapy. Carbohydr. Polym. 211, 360–369 (2019). 10.1016/j.carbpol.2019.01.010 30824100

[4] Turcheniuk, K. , et al.: Plasmonic photothermal cancer therapy with gold nanorods/reduced graphene oxide core/shell nanocomposite. RSC Adv. 6(2), 1600–1610 (2016). 10.1039/c5ra24662h

[5] Huang, X. , El‐Sayed, M.A. : Plasmonic photo‐thermal therapy (PPTT). Alexandria J. Med. 47(1), 1–9 (2011). 10.1016/j.ajme.2011.01.001

[6] Hong, Y. , et al.: Nanobiosensors based on localized surface plasmon resonance for biomarker detection. J. Nanomater. 2012(111), 1–13 (2012). 10.1155/2012/759830

[7] Chen, H. , et al.: Gold nanorods and their plasmonic properties. Chem. Soc. Rev. 42(7), 2679–2724 (2013). 10.1039/c2cs35367a 23128995

[8] Al‐Bakri, A.G. , Mahmoud, N.N. : Photothermal‐Induced antibacterial activity of gold nanorods loaded into polymeric hydrogel against *Pseudomonas aeruginosa* biofilm. Molecules 24(14), 2661–2680 (2019). 10.3390/molecules24142661 31340472 PMC6680386

[9] Park, K. , et al.: Engineering the optical properties of gold nanorods: independent tuning of surface plasmon energy, extinction coefficient and scattering cross‐section. J. Phys. Chem. C 118(11), 5918–5926 (2014). 10.1021/jp5013279

[10] Lankveld, D. , et al.: Blood clearance and tissue distribution of PEGylated and non‐PEGylated gold nanorods after intravenous administration in rats. Nanomedicine 6, 339–349 (2011). 10.2217/nnm.10.122 21385136

[11] Mackey, M.A. , et al.: The most effective gold nanorod size for plasmonic photothermal therapy: theory and in vitro experiments. J. Phys. Chem. B 118(5), 1319–1326 (2014). 10.1021/jp409298f 24433049 PMC3983380

[12] Link, S. , El‐Sayed, M.A. : Shape and size dependence of radiative, non‐radiative and photothermal properties of gold nanocrystals. Int. Rev. Phys. Chem. 19(3), 409–453 (2000). 10.1080/01442350050034180

[13] Qin, Z. , et al.: Quantitative comparison of photothermal heat generation between gold nanospheres and nanorods. Sci. Rep. 6(1), 29836–29848 (2016). 10.1038/srep29836 27445172 PMC4956767

[14] Baffou, G. , Quidant, R. , Girard, C. : Heat generation in plasmonic nanostructures: influence of morphology. Appl. Phys. Lett. 94(15), 153109–153111 (2009). 10.1063/1.3116645

[15] Yang, W. , et al.: Shape effects of gold nanoparticles in photothermal cancer therapy. Mater. Today Sustain. 13, 100078 (2021). 10.1016/j.mtsust.2021.100078

[16] Bucharskaya, A.B. , et al.: Plasmonic photothermal therapy: approaches to advanced strategy. Lasers Surg. Med. 50(10), 1025–1033 (2018). 10.1002/lsm.23001 30024039

[17] Jayabal, S. , et al.: A gold nanorod‐based localized surface plasmon resonance platform for the detection of environmentally toxic metal ions. Analyst 140(8), 2540–2555 (2015). 10.1039/c4an02330g 25738185

[18] Lázár, I. , Szabó, H.J. : Prevention of the aggregation of nanoparticles during the synthesis of nanogold‐containing silica aerogels. Gels 4(2), 55–64 (2018). 10.3390/gels4020055 30674831 PMC6209257

[19] Anderson, M.L. , et al.: Colloidal gold aerogels: preparation, properties, and characterization. Langmuir 15(3), 674–681 (1999). 10.1021/la980784i

[20] Moretti, L. , et al.: Plasmonic control of drug release efficiency in agarose gel loaded with gold nanoparticle assemblies. Nanophotonics 10(1), 247–257 (2021). 10.1515/nanoph-2020-0418

[21] Li, J. , et al.: Simple and rapid functionalization of gold nanorods with oligonucleotides using an mPEG‐SH/Tween 20‐assisted approach. Langmuir 31(28), 7869–7876 (2015). 10.1021/acs.langmuir.5b01680 26101941

[22] Lee, B.J. , et al.: Shaping nanoparticle diffusion through biological barriers to drug delivery. JCIS Open 4, 100025–100031 (2021). 10.1016/j.jciso.2021.100025

[23] Pratap, D. , et al.: Photothermal effects in small gold nanorod aggregates for therapeutic applications. Appl. Nanosci. 12(7), 2045–2058 (2022). 10.1007/s13204-022-02456-z

[24] Gong, B. , et al.: Thermo‐responsive polymer encapsulated gold nanorods for single continuous wave laser‐induced photodynamic/photothermal tumour therapy. J. Nanobiotechnol. 19(1), 41–55 (2021). 10.1186/s12951-020-00754-8 PMC7869504 33557807

[25] Meyer, S.M. , et al.: Size effects in gold nanorod light‐to‐heat conversion under femtosecond illumination. J. Phys. Chem. C 125(29), 16268–16278 (2021). 10.1021/acs.jpcc.1c03898

[26] Awan, U.A. , et al.: Doxorubicin‐loaded gold nanorods: a multifunctional chemo‐photothermal nanoplatform for cancer management. Beilstein J. Nanotechnol. 12, 295–303 (2021). 10.3762/bjnano.12.24 34012759 PMC8022204

[27] Bermúdez‐Jiménez, C. , et al.: Hydrogel‐embedded gold nanorods activated by plasmonic phototherapy with potent antimicrobial activity. Nanomedicine 22, 102093–102102 (2019) 31521833 10.1016/j.nano.2019.102093

[28] Yang, J. , et al.: Polypeptide‐engineered hydrogel coated gold nanorods for targeted drug delivery and chemo‐photothermal therapy. ACS Biomater. Sci. Eng. 3(10), 2391–2398 (2017). 10.1021/acsbiomaterials.7b00359 33445297

[29] Wang, X. , et al.: Understanding the photothermal effect of gold nanostars and nanorods for biomedical applications. RSC Adv. 4(57), 30375–30383 (2014). 10.1039/c4ra02978j

[30] Canpean, V. , Gabudean, A.M. , Astilean, S. : Enhanced thermal stability of gelatin coated gold nanorods in water solution. Colloids Surf. A Physicochem. Eng. Asp. 433, 9–13 (2013). 10.1016/j.colsurfa.2013.03.072

[31] Gans, R. : Über die Form ultramikroskopischer Goldteilchen. Ann. Phys. 342(5), 881–900 (1912). 10.1002/andp.19123420503

[32] Cong, B. , et al.: Gold nanorods: near‐infrared plasmonic photothermal conversion and surface coating. J. Mater. Sci. Chem. Eng. 2(1), 20–25 (2014). 10.4236/msce.2014.21004

[33] Wei, G. , et al.: Hairpin DNA‐functionalized gold nanorods for mRNA detection in homogenous solution. J. Biomed. Opt. 21(9), 097001–097009 (2016). 10.1117/1.jbo.21.9.097001 27604563

[34] Mbalaha, Z.S. , et al.: Synthesis of small gold nanorods and their subsequent functionalization with hairpin single stranded DNA. ACS Omega 4(9), 13740–13746 (2019). 10.1021/acsomega.9b01200 31497691 PMC6714599

[35] Lee, P.Y. , et al.: Agarose gel electrophoresis for the separation of DNA fragments. J. Vis. Exp. (62), (2012). 10.3791/3923 PMC4846332 22546956

[36] Latreille, P.‐L. , et al.: Spontaneous shrinking of soft nanoparticles boosts their diffusion in confined media. Nat. Commun. 10(1), 4294–4301 (2019). 10.1038/s41467-019-12246-x 31541104 PMC6754464

[37] Giannini, V. , et al.: Plasmonic nanoantennas: fundamentals and their use in controlling the radiative properties of nanoemitters. Chem. Rev. 111(6), 3888–3912 (2011). 10.1021/cr1002672 21434605

[38] Schuller, J. , et al.: Plasmonics for extreme light concentration and manipulation. Nat. Mater. 9(3), 193–204 (2010). 10.1038/nmat2630 20168343

[39] Davis, T.J. , Vernon, K.C. , Gómez, D.E. : Effect of retardation on localized surface plasmon resonances in a metallic nanorod. Opt. Express 17(26), 23655–23663 (2009). 10.1364/oe.17.023655 20052075

[40] Mbalaha, Z.S. : Gold Nanorod Based Nanoprobes for Biomedical Applications. Doctoral thesis. University of Strathclyde (2020)

[41] Freni, A. , et al.: Silica gel microfibres by electrospinning for adsorption chillers. Energy 187, 115971–115979 (2019). 10.1016/j.energy.2019.115971

[42] Noroozi, M. , et al.: Nanostructure of aerogels and their applications in thermal energy insulation. ACS Appl. Energy Mater. 2(8), 5319–5349 (2019). 10.1021/acsaem.9b01157

[43] Soto‐Reyes, N. , et al.: Effects of shape and size of agar gels on heating uniformity during pulsed microwave treatment. J. Food Sci. 80(5), E1021–E1025 (2015). 10.1111/1750-3841.12854 25827444

[44] Ramires, M.L.V. , et al.: Standard reference data for the thermal conductivity of water. J. Phys. Chem. Ref. Data 24(3), 1377–1381 (1995). 10.1063/1.555963

