# Supplementary material for: Photothermal effects of gold nanorods in aqueous solution and gel media: Influence of particle size and excitation wavelength
Source: IET Nanobiotechnol. 2022 Dec 21;17(2):103–11. doi: 10.1049/nbt2.12110 (PMC10116019; doi:10.1049/nbt2.12110)
Supplement: Supplementary file 1 — Supporting Information S1 [file NBT2-17-103-s001.docx]

**Supporting information**

To synthesize LGNRs, the seed solution was first prepared by adding 2.50ml of tetrachloroauric acid (0.001M) to 7.50ml of hexadecyltrimethylammonium (0.2M). After 2-3 minutes of stirring, 0.6ml of a freshly prepared ice-cold sodium borohydride (0.01M) were added and the mixture turned yellow-brown. The growth solution is prepared by adding 10ml of tetrachloroauric acid (0.001M) to 10ml of hexadecyltrimethylammonium (0.2M), then varying amount of silver nitrate (0.004M) solution were added to tune the surface plasmon resonance of the intended GNR, followed by the addition of 0.14ml ascorbic acid (0.0778M) which turns the growth mixture colourless. Then, 0.02ml of the seed solution was added to the growth mixture and incubated overnight to allow the growth of gold seeds to nanorods. The method of SGNRs synthesis was detailed in Reference 18 where a higher volume of seed solution was used in comparison with LGNRs synthesis as shown in table S1 and S2.

**Table S1.** The list of reagents used to prepare the seeds and the growth solutions of the LGNRs.

| Reagents | | HAuCl_4_ (0.001M; ml) | CTAB (0.2M; ml) | NaBH_4_ (0.01M; ml) | AgNO_3_ (0.004M; ml) | A.A (0.0778M; ml) | Seeds (ml) |
| --- | --- | --- | --- | --- | --- | --- | --- |
| Seeds solution | | 2.50 | 7.50 | 0.60 | - | - | - |
| Growth solution | LGNR-158 | 10.00 | 10.00 | - | 0.158 | 0.14 | 0.02 |
|  | LGNR-200 | 10.00 | 10.00 | - | 0.200 | 0.14 | 0.02 |
|  | LGNR-380 | 10.00 | 10.00 | - | 0.380 | 0.14 | 0.02 |

**Table S2.** The list of reagents used to prepare the seeds and growth solutions of the SGNRs.

| Reagents | | HAuCl_4_ (0.01M; ml) | CTAB (0.1M; ml) | NaBH_4_ (0.01M; ml) | AgNO_3_ (0.01M; ml) | HCl (1.0M; ml) | A.A (0.1M; ml) | Seeds (ml) |
| --- | --- | --- | --- | --- | --- | --- | --- | --- |
| Seeds solution | | 0.25 | 9.75 | 0.60 | - | - | - | - |
| Growth solution | G_9_S_1_-60 | 0.50 | 9.00 | - | 0.060 | 0.20 | 0.08 | 1.00 |
|  | G_9_S_1_-75 | 0.50 | 9.00 | - | 0.075 | 0.20 | 0.08 | 1.00 |
|  | G_9_S_1_-90 | 0.50 | 9.00 | - | 0.090 | 0.20 | 0.08 | 1.00 |


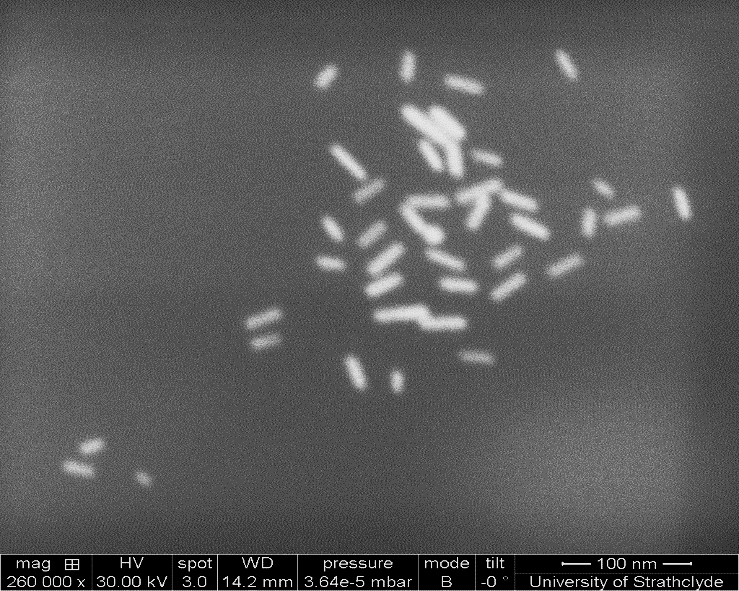

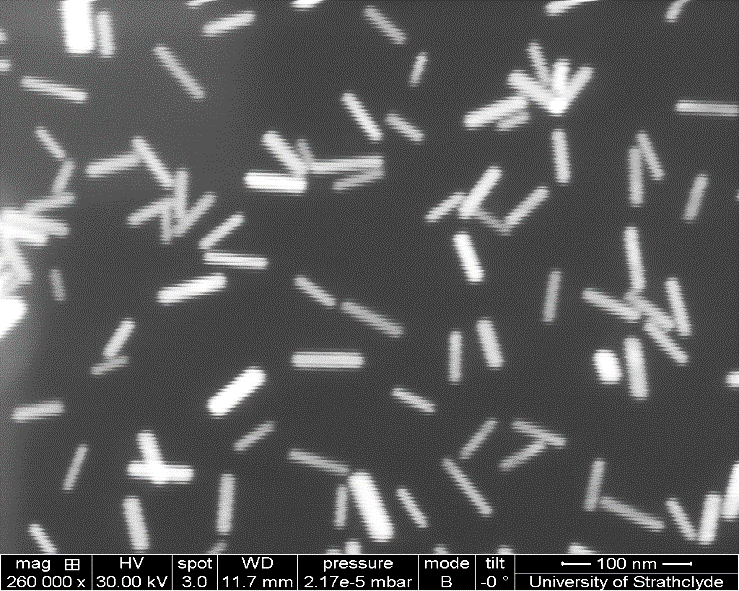


**a**

**b**

**Figure S1.** The SEM images; (a) S817 and (b) L816. The average length and diameter were extracted from SEM micrographs of over 100 particles for both SGNR and LGNR.

**Table S3.** The list of the parameters for calculating the heat generation of the SGNRs and the LGNRs. The absorption cross-section of the SGNRs and the LGNRs was calculated at their LSPR.

| Sample | S720 | S754 | S817 | L719 | L755 | L816 |
| --- | --- | --- | --- | --- | --- | --- |
| σ_abs_ (μm^2^) | 3.20E^-4^ | 3.79E^-4^ | 5.46E^-4^ | 2.73E^-3^ | 3.40E^-3^ | 3.96E^-3^ |
| C (mol/μm^3^) (x10^-24^) | 1.17 | 0.99 | 0.688 | 0.131 | 0.104 | 0.090 |
| N (μm^-3^) | 7.05E^-4^ | 5.96E^-4^ | 4.14E^-4^ | 7.89E^-5^ | 6.26E^-5^ | 5.42E^-5^ |
| V (μm^3^) | 3.30E^12^ | 3.30E^12^ | 3.30E^12^ | 3.30E^12^ | 3.30E^12^ | 3.30E^12^ |


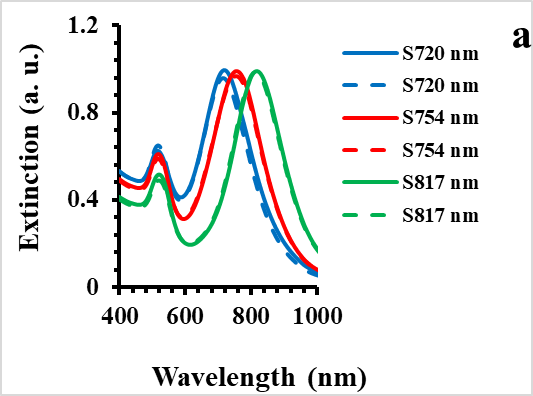

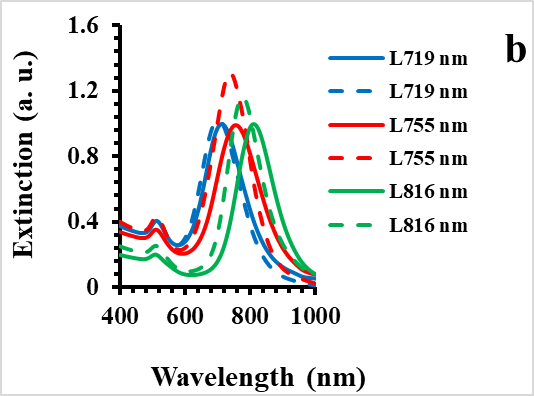


**Figure S2.** The UV-vis extinction spectra of the GNRs; (a) SGNRS and (b) LGNRs in water before (solid line) and after (dashed line) laser irradiation.
